# Assessing the predictive value of the healthy eating index in newly diagnosed atherosclerotic patients: a case-control study

**DOI:** 10.1186/s13104-025-07545-0

**Published:** 2025-10-29

**Authors:** Hanieh Abdi, Zohre Karamian, Kazem Hajiloo, Amin Sharifi

**Affiliations:** 1 Student Research Committee, Hamadan University of Medical Sciences, Hamadan, Iran; 2 Department of Nutrition and Food Hygiene, Hamadan University of Medical Sciences, Hamadan, Iran; 3 Nutrition Health Research Center, Institute of Health Sciences and Technology, Hamadan University of Medical Sciences, Hamadan, Iran

**Keywords:** Atherosclerosis, Diet, Nutrients, Healthy eating index, Heart disease risk factors, Healthy diet

## Abstract

**Objectives:**

Atherosclerosis, a chronic inflammatory condition characterized by arterial plaque buildup, contributes to cardiovascular disease-related morbidity and mortality. The Dietary Guidelines for Americans (DGA) inform nutrition policy, and the Healthy Eating Index (HEI) assesses diet quality based on its alignment with the DGA. This case-control study investigated HEI-2020 in newly diagnosed patients with atherosclerosis, compared to a healthy control group. Patients newly diagnosed with atherosclerosis comprised the case group, while gender- and age-matched volunteers served as the control group. Participants completed a validated 168-item food frequency questionnaire (FFQ). The Healthy Eating Index 2020 (HEI-2020) was calculated based on the FFQ data. Descriptive statistics and statistical tests such as the independent t-test and logistic regression were performed using SPSS software.

**Results:**

One hundred and three newly diagnosed atherosclerotic patients and an equal number of healthy controls were included. A significant difference was found in the mean HEI-2020 score between the case and control groups (*p* = 0.029). Logistic regression analysis indicated that HEI-2020 was a predictive factor for atherosclerosis development (*p* = 0.031), even after adjusting for BMI and physical activity (*p* = 0.035). Therefore, we found that HEI-2020 was a predictor of atherosclerosis incidence.

## Introduction

Atherosclerosis is widely recognized as one of the most important cardiovascular diseases (CVD) and is the leading cause of conditions such as myocardial infarction, stroke, and peripheral arterial disease. It is a chronic inflammatory disease in which the buildup of plaque in the walls of arteries leads to the narrowing and hardening of the arteries, which can reduce blood flow and cause clinical complications if plaques become unstable and rupture [1, 2]. It is estimated that atherosclerotic diseases account for more than half of all CVD-related deaths worldwide [3, 4]. 

A body of evidence has highlighted the significant role of healthy eating in the progression of atherosclerosis. Dietary factors can influence the vascular system by altering inflammation, oxidative stress, and active metabolites [5]. 

The Dietary Guidelines for Americans (DGA) serve as the cornerstone of nutrition policy for the U.S. government and underpin all federal dietary recommendations [6]. Since 1980, the DGA has undergone evidence-based reviews and updates every five years to reflect current evidence in nutritional science [7]. The Healthy Eating Index (HEI) is advocated as a tool to evaluate the holistic nutritional quality of dietary intakes by assessing the degree of concordance with DGAs. Reflecting periodic revisions to the DGA, several iterations of the HEI have been developed, with the most recent instantiation, HEI-2020, scrutinizing alignment with the 2020–2025 DGA recommendations [6, 8]. 

The HEI has demonstrated utility across diverse scientific inquiries, serving as a metric in surveillance studies to characterize population-level dietary quality, in epidemiological investigations to examine associations between nutritional quality and health outcomes, and in intervention-based research to assess the effects of dietary modifications [9]. Higher HEI scores have been associated with various chronic diseases, including cardiovascular diseases [10]. Studies have shown that adherence to the HEI, along with other dietary indices such as the DASH, is associated with a reduced risk of atherosclerosis [11]. In an Iranian study, higher HEI scores were associated with reduced levels of cardiovascular risk factors, which suggests that following a healthy diet, as measured by the HEI, may have cardioprotective effects [12]. 

This study aimed to investigate HEI-2020 in newly diagnosed atherosclerotic patients in Hamadan (Iran) and compare it with a healthy control group in 2024.

## Methods and materials

In this case-control study conducted in Hamadan City, Iran, in 2024, newly diagnosed patients with atherosclerosis were selected as the case group. The control group consisted of volunteers who were friends or neighbors of each patient, matched by gender and within a similar age range (± 5 years). The inclusion criteria for the case group required a definitive diagnosis of atherosclerosis [13] made within the previous three months, non-adherence to a prescribed dietary regimen, or significant changes in dietary habits during the three months preceding the study.

A validated food frequency questionnaire (FFQ) was used to obtain dietary information [14]. Nutritional data, including calorie intake, carbohydrates, protein, total fat, saturated fatty acids (SFA), monounsaturated fatty acids (MUFA), polyunsaturated fatty acids (PUFA), and dietary fiber, were analyzed using the ShaFA software [15], based on responses from the FFQ.

HEI-2020 was calculated [8, 16] using the nutritional data obtained from FFQ and the ShaFA software. Physical activity was assessed at two levels using two researcher-made questions (Data not shown). Statistical analysis was conducted using SPSS software, with logistic regression employed to adjust for confounding factors. The significance level was set at 0.05.

This study was conducted with the approval of the ethics committee at Hamadan University of Medical Sciences (ethics code: IR.UMSHA.REC.1401.790) and in accordance with the ethical principles outlined in the Declaration of Helsinki for all research involving human participants or materials. Informed written consent was obtained from all participants before their involvement in the study.

## Results

One hundred and three newly diagnosed atherosclerotic patients and the same number of healthy controls were included.

There was a significant difference in the mean HEI-2020 between the two groups (*p* = 0.029). A significant difference was also observed in the mean HEI-2020 scores between the atherosclerosis group and the control group among men (*p* = 0.012), but not among women (*p* = 0.493) (Table 1).

**Table 1:** Descriptive statistics and comparisons of HEI-2020 between atherosclerotic patients and healthy control groups

		Men		Women		Total	
		Controls (*n* = 52)	Cases (*n* = 53)	Controls (*n* = 51)	Cases (*n* = 50)	Controls (*n* = 103)	Cases (*n* = 103)
HEI-2020	Mean	52.65	47.87	51.41	49.86	52.04	48.83
	SD	9.94	9.17	12.53	9.99	11.26	9.58
	Median	50.57	46.55	51.98	47.95	50.67	47.57
	IQR	14.00	12.43	19.01	17.99	17.6	14.48
	Min	33.61	32.84	28.16	30.30	28.16	30.30
	Max	82.62	71.26	73.78	72.23	82.62	72.23
	p	0.012		0.493		0.029	

In the logistic regression analysis, HEI-2020 was a significant predictor of developing atherosclerosis (OR: 0.971; 95% CI 0.945–0.997; *p* = 0.031), even after adjusting for BMI and physical activity level (OR: 0.945; 95% CI 0.945–0.998; *p* = 0.035).

## Discussion

In this case-control study, we found that higher HEI-2020 was a protective factor against the incidence of atherosclerosis, even after adjusting for BMI and physical activity level.

HEI is a tool used to assess how well a set of foods aligns with dietary guidelines, such as the DGA. Higher HEI scores indicate better adherence to these guidelines, which in general emphasize the consumption of fruits, vegetables, whole grains, and healthy fats, while limiting the intake of sodium, saturated fats, and added sugars [6, 8]. 

The HEI’s focus on nutrient-rich foods contributes to improvements in several CVD risk factors. For example, increased consumption of fruits and vegetables is associated with lower blood pressure and improved lipid profiles. Whole grains contribute to better glucose control and reduced inflammation. Limiting saturated fats and sodium helps maintain healthy cholesterol levels and blood pressure [17]. HEI evaluates overall dietary patterns rather than individual nutrients or foods. This holistic approach recognizes the complex relationships between various dietary components and their cumulative effect on overall health. Similarly, dietary recommendations for preventing atherosclerosis emphasize the consumption of plant-based foods, limiting foods of animal origin, and reducing salt intake [18]. 

An evaluation of the Dietary Patterns Methods Project (DPMP), which incorporated the HEI-2010, and the Alternative HEI-2010 demonstrated that higher scores were associated with reductions in CVD incidence and mortality rates [19]. Similarly, in the NHANES study, HEI-2015 was associated with the risk of abdominal aortic calcification in a U-shaped pattern [20]. Additionally, an analysis of ARIC study data showed that higher adherence to the DGA 2015–2020 guidelines was associated with lower risks of CVD incidence, CVD mortality, and all-cause mortality [21]. 

A case-control study in Ahvaz (Iran) found that a higher HEI score may be associated with a reduced risk of atherosclerosis [22]. Moreover, an analytical cross-sectional study on a sample of older adults in Isfahan (Iran) suggested a relationship between HEI score and some cardiovascular risk factors, including HOMA-IR, FBS, hs-CRP, and HDL-C levels [12]. Our study results align with those of previous research, highlighting that adherence to the DGA may play a protective role in reducing the incidence of atherosclerosis.

Low-grade chronic systemic inflammation and oxidative stress are considered fundamental contributors to the development of atherosclerosis, as they can trigger the formation of vascular plaques [23]. Adhering to a healthy diet is believed to halt or delay this process. For instance, increasing antioxidant intake by consuming more fruits and vegetables, avoiding processed and sweetened foods, and reducing saturated and trans fatty acids has been associated with lower levels of inflammation and oxidative stress, which in turn may result in a reduced incidence of cardiovascular disease [24, 25]. 

## Limitations

The limitations of our study include the inherent measurement error associated with self-reported FFQs, which may have resulted in an attenuation or distortion of the real associations, as well as recall bias, over-reporting, and under-reporting, common issues in case-control studies [26]. However, to mitigate this potential error, the FFQs were administered in person by trained interviewers. Furthermore, the healthy control group did not undergo systematic screening to exclude underlying conditions, and the controls were not randomly selected from the entire target population, which may have introduced selection bias. Our study had several strengths, including the use of trained interviewers to administer the FFQ and the inclusion of new-case patients to minimize the influence of dietitian- or physician-prescribed changes in dietary habits.

## Data Availability

Available from the corresponding author on reasonable request.
